# MPXV: Update on Morphological and Morphogenesis Aspects Through Transmission and Scanning Electron Microscopies and 3D Reconstruction

**DOI:** 10.1002/jmv.70180

**Published:** 2025-01-18

**Authors:** Debora Ferreira Barreto‐Vieira, Milene Dias Miranda, Marcos Alexandre Nunes da Silva, Andressa Santos de Almeida, Ana Luisa Teixeira de Almeida, Derick Mendes Bandeira, Vivian Neuza S. Ferreira, Alice Santos Rosa, Wendell Girard‐Dias, Bráulio Soares Archanjo, Ortrud Monika Barth

**Affiliations:** ^1^ Laboratório de Morfologia e Morfogênese Viral Instituto Oswaldo Cruz, Fundação Oswaldo Cruz‐Fiocruz Rio de Janeiro Brazil; ^2^ Plataforma de Microscopia Eletrônica Rudolf Barth Instituto Oswaldo Cruz, Fundação Oswaldo Cruz‐Fiocruz Rio de Janeiro Brazil; ^3^ Núcleo de Laboratórios de Microscopia Instituto Nacional de Metrologia, Qualidade e Tecnologia Rio de Janeiro Brazil

**Keywords:** Mpox, MPXV, scanning electron microscopy, transmission electron microscopy, tridimensional reconstruction

## Abstract

An unprecedented global outbreak caused by the monkeypox virus (MPXV) prompted the World Health Organization to declare a public health emergency of international concern on July 23, 2022. Therapeutics and vaccines for MPXV are not widely available, necessitating further studies, particularly in drug repurposing area. To this end, the standardization of in vitro infection systems is essential. The most robust in vitro studies on poxviruses concern the Vaccinia virus, and there are significant gaps in understanding the replicative cycle of MPXV. Herein, we conducted ultrastructural studies using transmission and scanning electron microscopies and 3D reconstruction to describe and elucidate the step‐by‐step morphogenesis of MPXV. Vero cells, derived from the kidney lineage of Cercopithecus aethiops monkeys, were infected with a strain isolated from an oropharyngeal swab of a patient with suspected Mpox, collected during an observational cohort study conducted between June 12 and August 19, 2022, in Rio de Janeiro, Brazil. Infected Vero cells exhibited several morphological alterations, including cell lysis plaque formation, nuclei with altered chromatin profiles, thickening of the rough endoplasmic reticulum (RER), presence of myelin figures, disorganization of mitochondrial cristae, and the formation of a granular and fibrous matrix (viral factory) surrounded by mitochondria and RER cisternae in a perinuclear space. Viral entry into cells occurred via endocytosis MPXV particles were observed adhering to cytoskeletal filaments, and viral progeny extrusion occurred through exocytosis. This article presents novel data on the morphogenesis of MPXV that have not been previously documented in the literature.

## 1 Introduction

The monkeypox virus (MPXV), the causative agent of monkeypox disease (Mpox) [1], belongs to the *Poxviridae* family and the *Orthopoxvirus* genus, which includes other medically significant viruses such as Cowpox virus, Vaccinia virus, and Variola virus (VARV) [2]. More than 60 years after the first identification of human Mpox [3], the 2022 outbreak presented an unprecedented scenario, affecting numerous locations globally. While many of these locations are in Europe, the most severely impacted countries in terms of cases and mortality were the USA and Brazil, accounting for over 40% of the total cases worldwide. Out of a significant global total of 94,274 cases, the USA reported 32,125 cases and 58 deaths, whereas Brazil reported 10,967 cases and 16 deaths [4].

The emergence of international concern regarding Mpox coincided with the detection of cases in the United States and Great Britain in 2021 and the following year [5, 6]. Since then, the number of cases has risen, with more countries reporting the disease. In July 2022, the World Health Organization (WHO) declared the Mpox outbreak a public health emergency of international concern [7]. This event was marked by unusual clinical features [8], with the spread of MPXV being exceptional, as over 94% of the locations reporting cases had not historically documented the virus circulation [4, 9]. Moreover, the high prevalence of cases associated with sexual contact transmission [10] highlights a new perspective on the epidemiological behavior of Mpox.

Recent genomic analyses have provided deeper insights into the molecular epidemiology of the MPXV. A study by Forni et al. (2023) sequenced the complete genomes of hMPXV1 in five cases from Italy, confirming the presence of the predominant epidemic lineage circulating during the 2022 outbreak. These cases, reported between June and July 2022, exhibited the typical course of infection associated with hMPXV1. The sequencing data supported the hypothesis that this epidemic lineage was responsible for the rapid spread observed in nonendemic regions, reflecting its adaptation and the new transmission dynamics of the virus [11]. The identification of this specific lineage reinforces the importance of genomic surveillance in tracking viral evolution and informing public health strategies during outbreaks.

The cessation of smallpox immunization, caused by the VARV, is a major factor linked to Mpox outbreaks in nonendemic regions, along with increased human mobility in a globalized world [12, 13]. VARV shares not only genome sequence similarity with MPXV, but also clinical features. Misdiagnosis remains a concern as smallpox, caused by VARV, and chickenpox, caused by the Varicella zoster virus, are part of the differential diagnosis [14]. It is crucial to diagnose the epidemiological aspects and specificities observed in outbreaks and patient anamnesis. The 2022 Mpox outbreaks included many cases of genital lesions [15], underscoring the importance of understanding new transmission settings of the etiological agent. Diagnostic methods for case confirmation include polymerase chain reaction (PCR) techniques, anti‐orthopoxvirus immunoglobulin M (IgM), and immunoglobulin G (IgG) detection, and direct visualization using transmission electron microscopy (TEM) with clinical specimens, preferably skin lesion isolates [16].

As mentioned, TEM is one of the techniques used for MPXV detection and is the only one that provides direct visualization of virus particles [17]. The challenges associated with TEM include the high costs of reagents and equipment and the need for trained professionals to process samples within strict biosafety and quality standards and perform the analysis to identify the etiologic agent [18]. Some studies consider TEM obsolete for routine diagnosis, as it does not meet the demands of epidemic situations where multiple diagnoses are required in locations lacking such resources [19].

However, TEM is invaluable for epidemiological events that require rapid identification of unknown agents, as it does not require prior knowledge about the agent for testing. Unlike other techniques that confirm specific agents using specific primers or antisera, TEM detection only requires prior negative staining of the sample, a fast and universal technique allowing analysis in just 10 min [20]. TEM has a long history with *Orthopoxviruses*, being used for rapid and differential diagnosis of smallpox since the 1960s [21]. Furthermore, TEM currently provides high‐resolution images essential for understanding the morphogenesis, pathogenesis, and transmission of etiological agents. When combined with structure labeling, cryo‐preparation, and 3D reconstruction, TEM can expand the range of possible analyses [22, 23].

The primary studies on poxviruses using electron microscopy have predominantly focused on the vaccinia virus, which serves as an experimental model due to its accessibility and relative safety in laboratory settings. While these studies have provided significant insights into the morphology and some phases of the replicative cycle of poxviruses, many aspects of this process remain obscure. Electron microscopy has enabled the identification of the basic forms of viral particles and some structural events during replication, such as the formation of the viral core and the maturation of virions [24]. However, the detailed mechanisms of assembly, release, and viral interactions with the host cell machinery still require further elucidation, particularly in emerging poxvirus species such as MPXV [11, 25]. Studies on the vaccinia virus have been fundamental to initial understanding, but the replicative cycle of other poxviruses remains underexplored. This highlights the need for further research to investigate the unexplored stages of the replicative cycle of these viruses.

Although MPXV is well‐known, gaps remain in our understanding of its transmission, morphogenesis, and pathogenesis. Recognizing these challenges, we utilized advanced electron microscopy techniques, particularly TEM, to present detailed aspects of MPXV's morphogenesis in a didactic manner.

In conclusion, the application of TEM in the study of viruses, including MPXV, has provided crucial insights into the virus's morphogenesis, offering a comprehensive understanding that is critical for developing effective diagnostic and therapeutic strategies. Despite its limitations, TEM remains an essential tool in virology, capable of delivering rapid and precise identification of viral pathogens, thereby playing a vital role in managing and controlling outbreaks.

## 2 Materials and Methods

### 2.1 MPXV Strain

The MPXV strain employed in this study was sourced from the National Reference Laboratory for Diagnosis in MPXV (Laboratório de Vírus Respiratórios, Exantemáticos, Enterovírus e Emergências Virais, Instituto Oswaldo Cruz, Fiocruz, Rio de Janeiro, Brazil) [26, 27]. This strain was isolated from an oropharyngeal swab obtained from a patient with suspected Mpox, as part of an observational cohort study conducted between June 12 and August 19, 2022, in Rio de Janeiro, Brazil. Our research team conducted the virus isolation and titration [27]. For virus isolation, Vero cells (Cercopithecus aethiops monkey kidney cells; BCRJ code: 0245) were seeded in a 12‐well plate (2.0 × 10^5^ cells/well) and incubated with 150 µL/well of a patient strain in Dulbecco's Modified Eagle Medium (DMEM; high glucose with sodium pyruvate, Life Technologies, Grand Island, NY), supplemented with 100 U/mL penicillin and 100 mg/mL streptomycin (Sigma‐Aldrich, Burlington, MA), and maintained at 37°C in a 5% CO₂ atmosphere. Following a 1‐h incubation period, 850 µL/well of fresh medium supplemented with 2% Fetal Bovine Serum (FBS; Life Technologies, South American, Brazil) was added. The cell monolayers were inspected daily under light microscopy for the development of cytopathic effects (CPE) up to 6 days postinfection. Virus titration was conducted using the 50% tissue culture infectious dose (TCID₅₀) method. Vero cell monolayers (1.5 × 10^4^ cells/well) were incubated with 50 μL of supernatants from previously infected cultures, initially used for virus isolation, at serial 10‐fold dilutions (10²–10⁵) with 10 replicates. After a 1‐h incubation at 37°C in a 5% CO₂ atmosphere, 50 μL of medium (DMEM high glucose, supplemented with sodium pyruvate, 100 U/mL penicillin, 100 mg/mL streptomycin, and 2% FBS) was added. Following a 72 h incubation period, CPE were observed via light microscopy, and 100 μL of 4% formaldehyde was added to fix the cells. After 3 h, the fixative was removed, and cell monolayers were stained with a 0.04% crystal violet solution in 20% ethanol for 1 h.

Viral detection in the clinical sample and in the cell culture isolate was performed by qRT‐PCR by the groups of Drs. Edson Silva and Thiago Moreno Lopes e Souza, respectively, using previously published methodology [24]. The virus title was 10^4.7^ TCID_50/mL_ with a cycle threshold (Ct) equal to 30. The study adhered to the Declaration of Helsinki guidelines and received approval from the Ethics Review Board at the Instituto Nacional de Infectologia, Fiocruz (CAAE # 61290422.0.0000.5262). Access to the genetic material was authorized by the National System for the Management of Genetic Heritage and Associated Traditional Knowledge (Sistema Nacional de Gestão do Patrimônio Genético e do Conhecimento Tradicional Associado ‐ SisGen; approval number: A19C586). All experimental procedures were performed in a biosafety level 3 (BSL‐3) laboratory, following the biosafety guidelines of the World Health Organization and the Biosafety in Microbiological and Biomedical Laboratories 6th Edition (CDC/NIH) [28].

### 2.2 Cells Infection for Ultrastructural Analysis by Transmission Electron Microscopy (TEM)

Prior to infection, monolayers of *Cercopithecus aethiops* monkey kidney cells (Vero, BCRJ code: 0245) were maintained in DMEM Medium supplemented with 10% FBS and 100 U/mL penicillin‐streptomycin (1× Pen‐Strep) at 37°C and 5% CO_2_. All cell culture reagents were procured from Gibco (Waltham, MA). For the infection procedure, the monolayers were washed twice with phosphate‐buffered saline (PBS), inoculated with a multiplicity of infection (MOI) of 0.1 in nonsupplemented DMEM, and incubated for 1 h at 37°C to allow for virus adsorption. Following adsorption, the inoculum was removed, and the cells were maintained at 37°C in DMEM supplemented with 2% FBS and 1× Pen‐Strep. Non‐infected control cultures were prepared using supplemented DMEM. Monolayers were inspected daily under a light microscope for the observation of CPE until 6 days post‐infection. All procedures were conducted in a BSL‐3 laboratory, following WHO biosafety guidelines (CDC/NIH) [28].

### 2.3 Sample Processing for TEM Analysis

For TEM analysis, infected monolayers of *Cercopithecus aethiops* monkey kidney cells (Vero, BCRJ code: 0245) were trypsinized at 6 days post‐infection, while non‐infected cells cultured for the same duration served as controls. The resulting cell suspensions were fixed and post‐fixed by immersion in a 2.5% glutaraldehyde solution in sodium cacodylate buffer (0.2 M, pH 7.2) and in 1% buffered osmium tetroxide, respectively. Following fixation and post‐fixation, the samples were dehydrated through immersion in increasing concentrations of acetone (15%, 30%, and 50% for 15 min each; 70% in 1% uranyl acetate for 30 min; 90% for 5 min; and twice in 100% for 10 min). Subsequently, the cells were embedded in epoxy resin and polymerized at 60°C for 3 days [29, 30]. Ultrathin sections (50–70 nm) were then obtained from the resin blocks using an ultramicrotome equipped with diamond knives. The sections were collected on uncoated copper grids with a mesh size of 300 and examined using a Cs‐corrected FEI Titan 80–300 (ThermoFisher Scientific, Waltham, MA) and a Hitachi HT 7800 (Hitachi, Tokyo, Japan) transmission electron microscope. In the FEI Titan, images were acquired in scanning transmission electron microscopy (STEM) mode, utilizing a high‐angle annular dark field (HAADF) detector, operating at 80 kV high tension, with a convergence angle of 19 mrad and a probe size of approximately 0.1 nm, a beam current of 100 pA, a dwell time ranging from 2 to 8 μs, and a resolution of 2048 × 2048 pixels per image.

### 2.4 Focused Ion Beam Scanning Electron Microscopy (FIB‐SEM) and 3D Modeling

FIB‐SEM image was acquired with a Helios Nanolab 650 electron microscope (ThermoFisher Scientific, Waltham, MA). The epoxide‐embedded sample (block) of Vero cells 6 days post‐infection with MPXV, prepared for TEM as described above, was mounted on a support stub, coated with a 10 nm layer of gold, and transferred to the microscope chamber. The block surface was oriented perpendicularly to electron beam. The area of interest was exposed by FIB milling of a U‐shaped trench. Milling conditions were 30 kV acceleration voltage, a beam current of 16 nA (for coarse milling), and 2 nA for polishing. One thousand two hundred sections were taken under the FIB and performed at 20 nm thickness with an ion beam current of 1 nA. Imaging of the block surface was carried out at 5 kV using in‐lens mode with a retractable low‐voltage high‐contrast detector (vCD) backscattered electron detector and 5.4 nm pixel size.

The image series were aligned using IMOD software [31], and the structures of interest were manually segmented to produce 3D models using IMOD and Amira (Thermo Fisher Scientific).

### 2.5 Helium Ion Scanning Electron Microscopy (SEM)

For analysis in helium ion microscopy, infected and non‐infected control monolayers were grown on sterile glass coverslips of 13 mm^2^ (Glasscyto). *Cercopithecus aethiops* monkey kidney cells (Vero, BCRJ code: 0245) in DMEM‐Higher medium (Gibco) were seeded in this 24 well plate (5 × 10^4^ cells/well) to adhere. After 24 h, cells were inoculated with a MOI of 0.1 for 1 h at 37°C and 5% CO_2_. Then, the supernatants were removed, and the cells were incubated with 400 µL of DMEM‐Higher medium with 2% FBS (Gibco) for 72 h at 37°C and 5% CO_2_. Uninfected cells were prepared as infected cells and colected at 72 h of cultivation (cell control). Given the 72 h, the supernatants the infected and no infected cultures were harvested, the cell monolayers were washed one time with PBS1X, and fixed with 300 µL of 2.5% glutaraldehyde in sodium cacodylate buffer (0.2 M, pH 7.2) (Electron Microscopy Sciences), dehydrated in ethanol, submitted to critical‐point‐drying and coated with a 15 nm carbon layer. The cells were analyzed in an Orion NanoFab Helium Ion microscope (Zeiss, Baden‐Württemberg, Oberkochen, Germany) equipped with a flood gun and an Everhart–Thornley secondary electrons detector, flood gun is used to allow analyzing the cells without any conductive coating. Images were collected at 30 kV high tension, around 0.8 pA beam current, 32 up to 256‐line average, 1 µs dwell time, 100 µs flood gun time, and 2048 × 2048 pixels per image [32].

## 3 Results

### 3.1 Cellular Alterations

**Figure 1:**
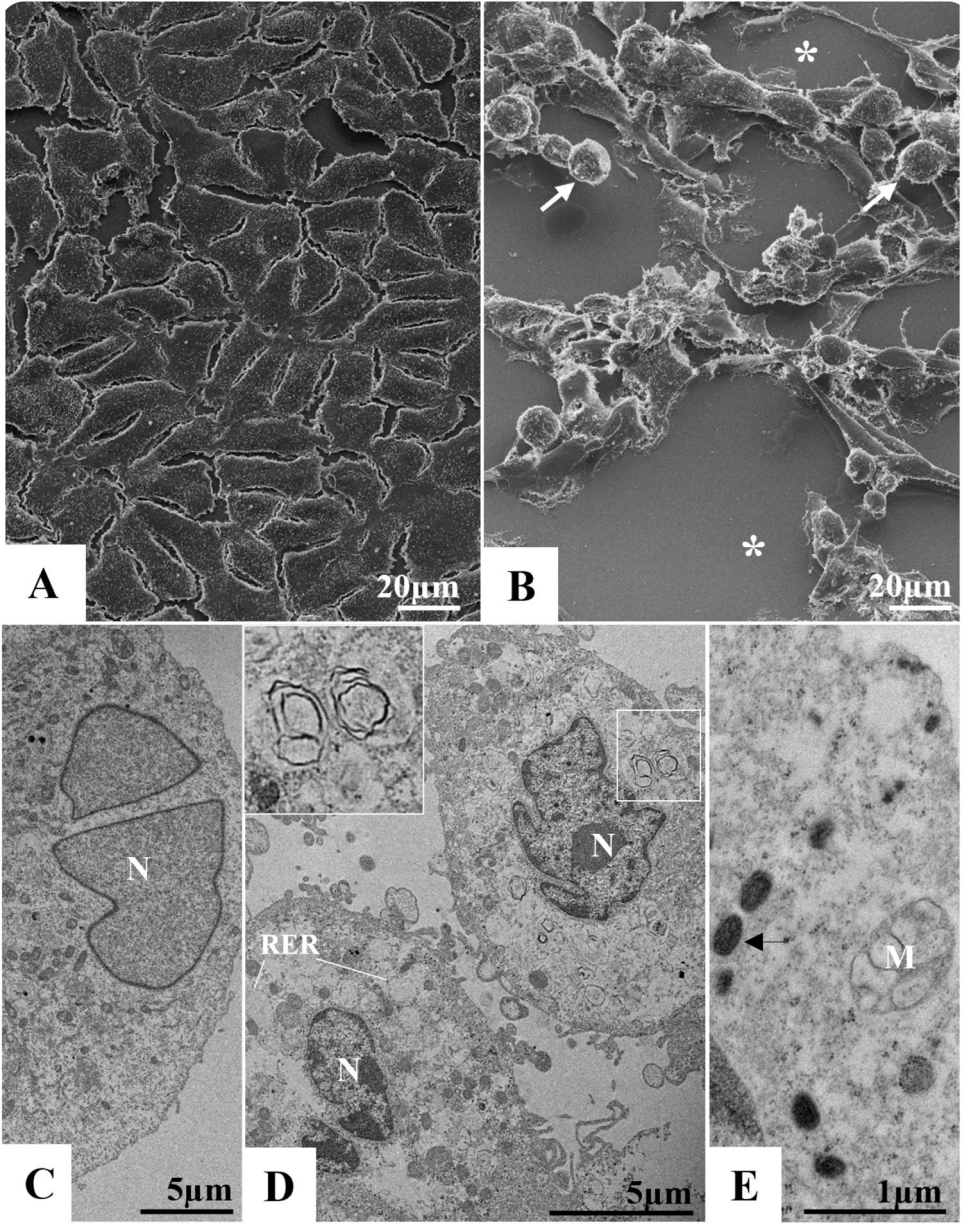
Alterations of Vero cell monolayer 6 days post‐infection with monkeypox virus (MPXV). (A and C) Uninfected cells (control cells). (B) Rounding of cells (white arrow), cell lysis plaque formation (*). (D) Thickening of rough endoplasmic reticulum (RER), presence of myelin figures (inset), nuclei (N) presenting changes in chromatin profile. (E) Disorganization of mitochondrial crests (M). Mpox particles (black arrow). (A and B) SEM images, (C–E) transmission electron microscopy (TEM) images.

**Figure 2:**
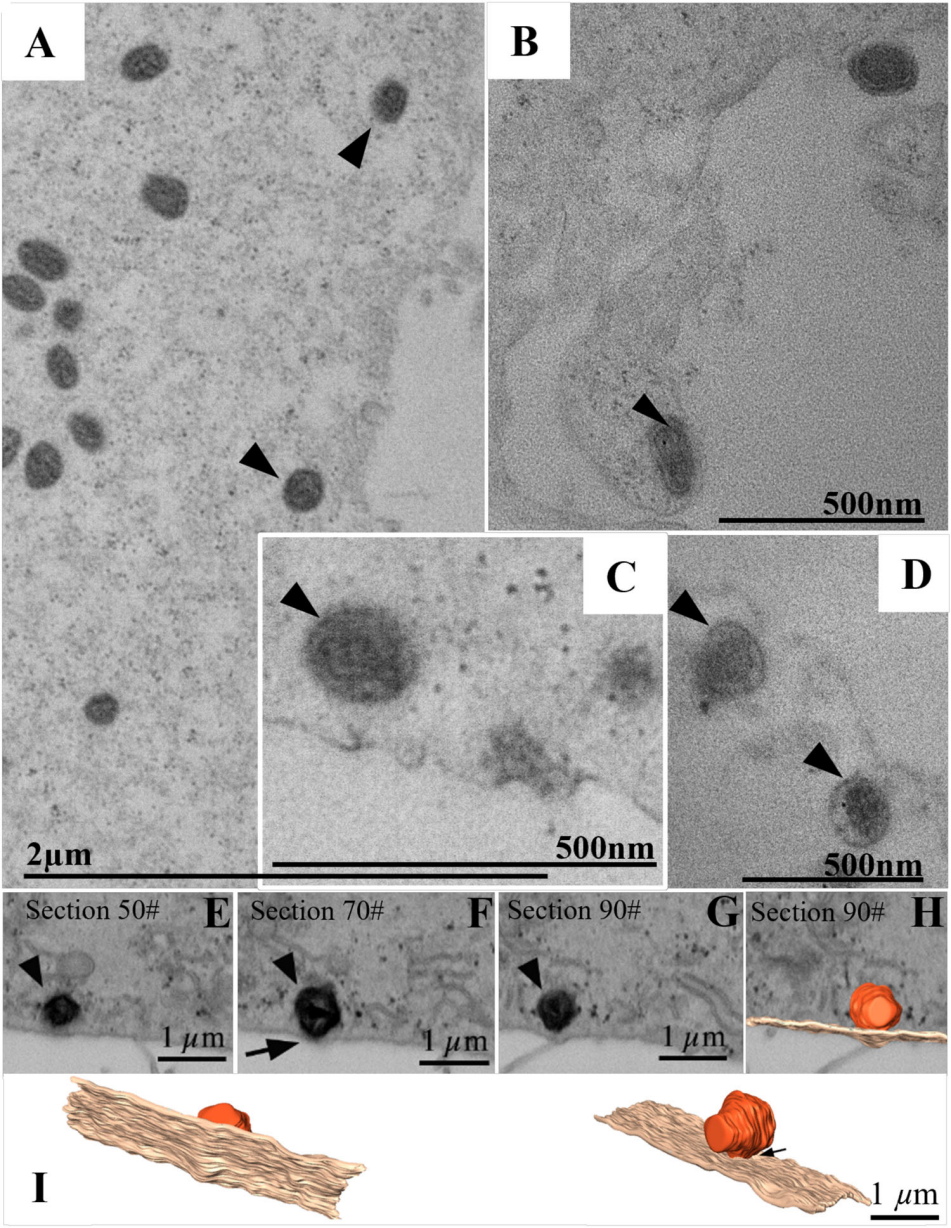
Internalization of monkeypox virus (MPXV) particles via endocytosis. (A–D) Transmission electron microscopy (TEM) images of Vero cells displaying viral particles at the cell periphery (A) and undergoing endocytosis (B–D) (arrowhead). (E–I) 3D Modeling of MPXV particles' entry by endocytosis. (E–H) FIB‐SEM images of the block surface sequence showing the entry of the MPXV particle. The arrow points a region of close contact between the MPXV particle and cell membrane. (H and I) Combination of a block surface image and the 3D model of structures of interest at different angle view. Orange (MPXV particles), light brown (cell membrane). MPXV particles (arrowhead). See video in Supplementary Material (Video S1).

**Figure 3:**
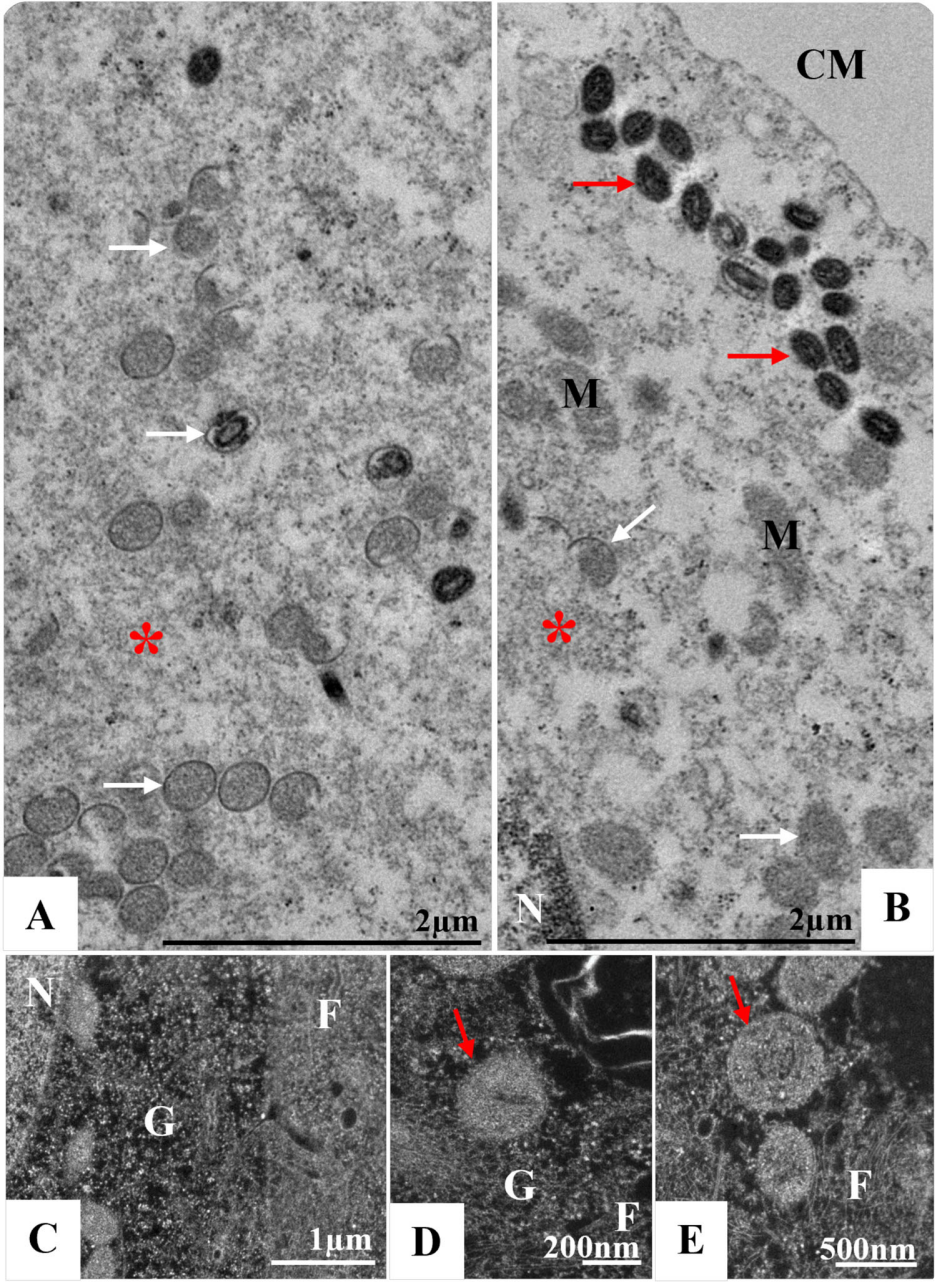
Replicative complex of monkeypox virus (MPXV) in Vero cells. Note MPXV particles in formation with different maturation profiles (white arrows) in a granular (asterisk) and fibrous (F) matrix surrounded by mitochondria (M) and rough endoplasmic reticulum (RER) cisterns. Nuclei (N), cell membrane (CM), virions (red arrow). Transmission electron microscopy (TEM) images.

**Figure 4:**
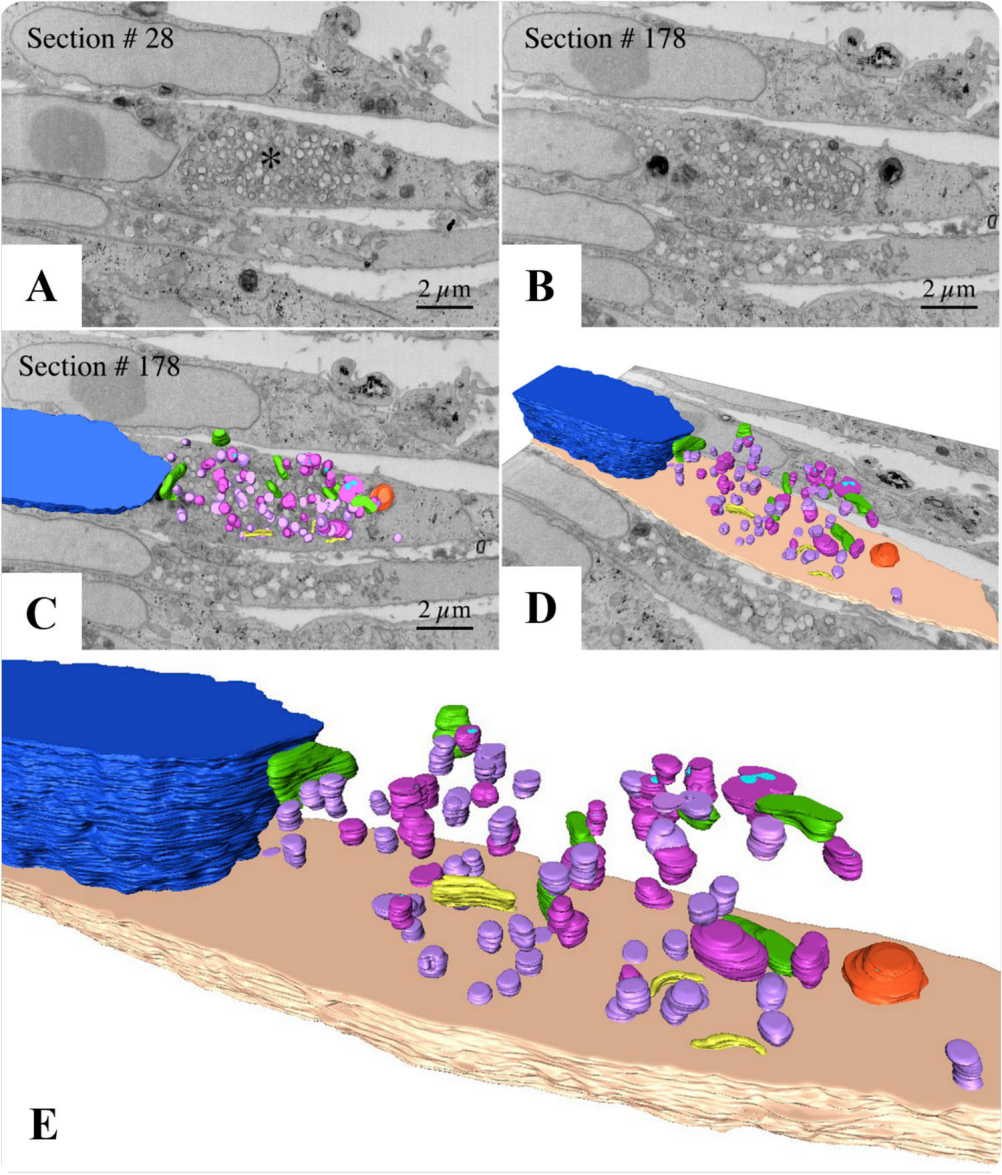
3D modeling of the replicative complex of monkeypox virus (MPXV) in Vero cells. (A and B) FIB‐SEM sequence images of the block surface showing infected cells and the viral factory area (asterisk). (C and D) Combination of a block surface image and the 3D model of structures of interest. (E) Enlarged view of cellular compartments involved in MPXV replication. Dark blue (nucleus), orange (virion), green (mitochondria), yellow (rough endoplasmic reticulum), violet (empty vesicles), purple (immature MPXV particles), light blue (viral DNA). A and B: FIB‐SEM. See video in Supplementary Infromation (Video S2).

**Figure 5:**
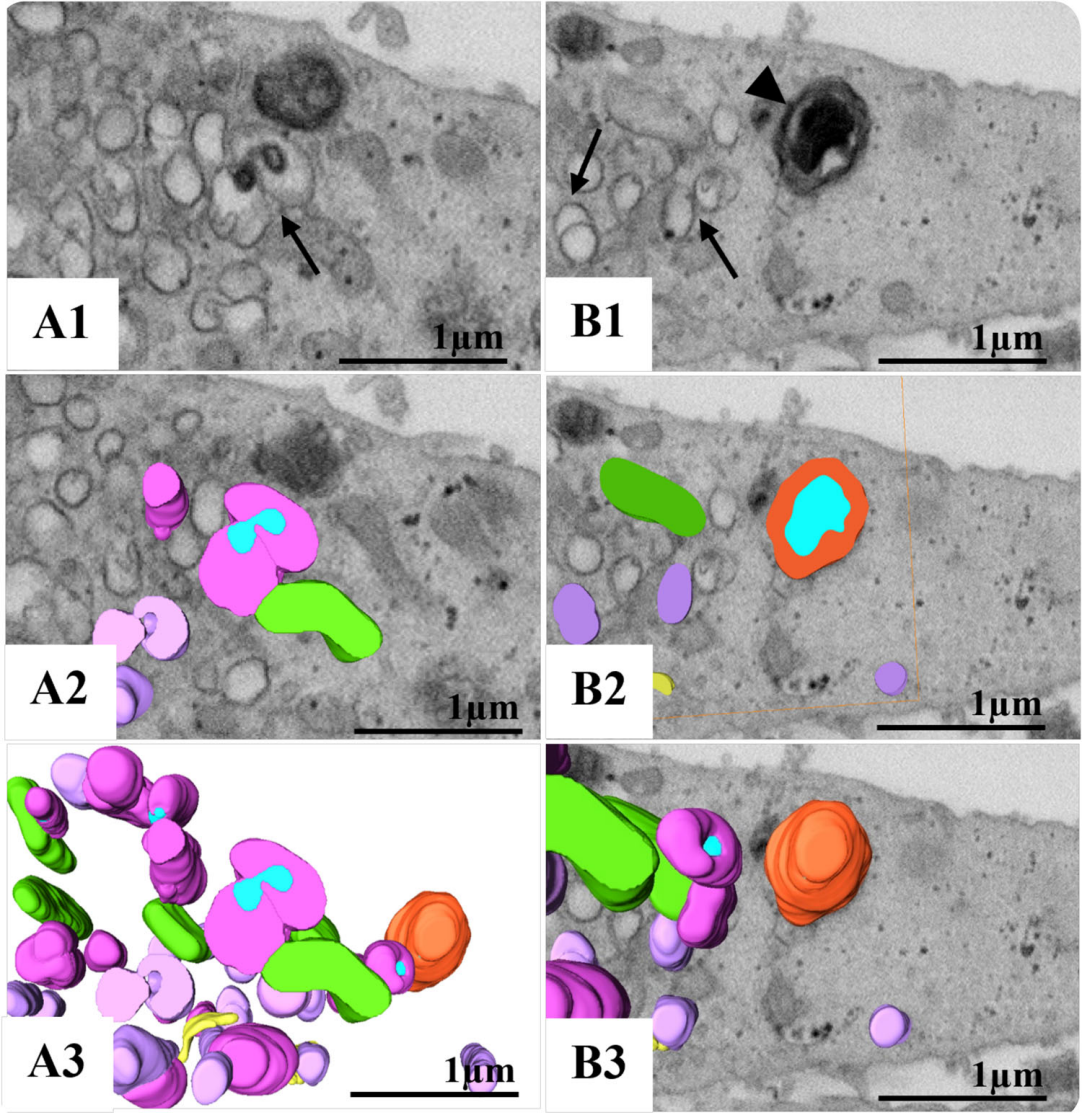
3D model of mature (arrowhead) and immature (arrow) monkeypox virus (MPXV) particles in the replicative complex. (A1 and B1) FIB‐SEM image of the block surface showing MPXV particles. (A2, B2, B3) Combination of a block surface image and the 3D model of structures of interest. (A3) 3D model. Orange (virion), green (mitochondria), yellow (rough endoplasmic reticulum), violet (empty vesicles), purple (immature MPXV particles), light blue (viral DNA). A1 and B1: FIB‐SEM.

Vero cells infected with MPXV exhibited a range of alterations when compared to uninfected cells. The control culture (uninfected cells) demonstrated a confluent monolayer characterized by rarely lobed nuclei and mitochondria with intact cristae (Figure 1A,C). In the infected cells, the predominant alterations included cell rounding, the formation of lysis plaques (Figure 1B), nuclei displaying alterations in chromatin profiles, thickening of the rough endoplasmic reticulum (RER), presence of myelin figures, and cytoplasmic rarefaction (Figure 1D). Additionally, there was disorganization of mitochondrial cristae (Figure 1E) and the presence of a granular and fibrous matrix surrounding the mitochondria and RER cisternae in the perinuclear space. Virions exhibiting the characteristic morphology of poxviruses—distinguished by their large, oval to brick‐shaped structures—were observed at various points within the cytosol (Figures 2A–G, 4A,B, 5B1, 6A–F, 7, 8A,A1–C1, 9B–E), along with immature particles embedded within the granular and fibrous matrix (Figures 3A–F, 5A1,B1, 9A).

### 3.2 Virus Particle Internalization

In our analyses utilizing TEM, FIB‐SEM and three‐dimensional modeling, we observed that the particles of the MPXV are internalized via endocytosis (Figure 2A–I, Supplementary Material [Video S1]). A total of ninety infected cells were examined using TEM with the aim of detecting MPXV particles during the internalization process. Among these, endocytosis was predominantly observed in 20 of the 90 cells analyzed. Numerous virions were identified within vesicles located at the cell periphery. These quantitative findings underscore the significance of endocytosis as a mechanism for MPXV entry into Vero cells, thereby providing a robust foundation for our conclusions.

### 3.3 Morphogenesis of MPXV Virus in Vero Cells

**Figure 6:**
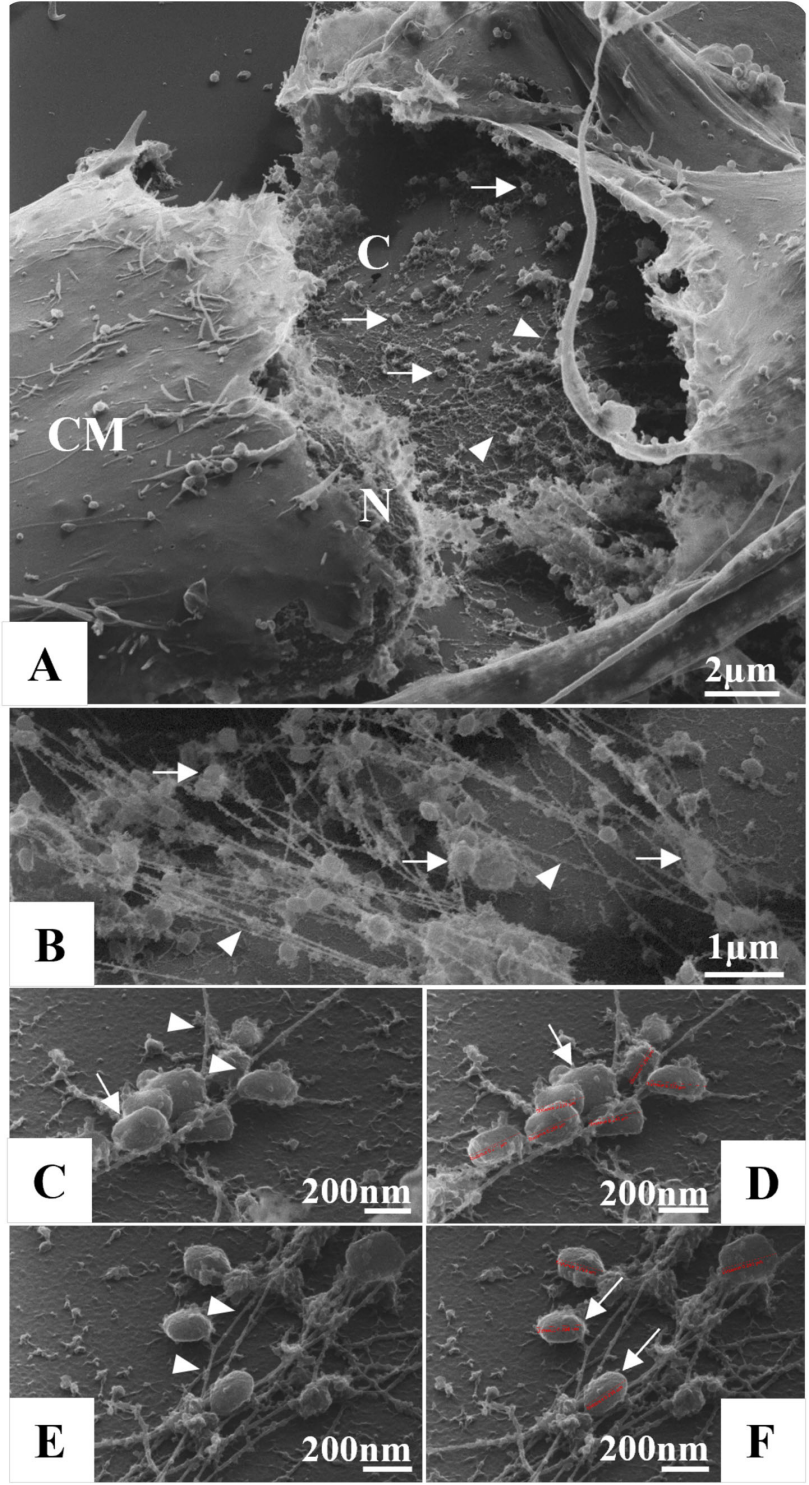
Interaction of monkeypox virus (MPXV) particles with the cell's cytoskeleton. Cytoskeletal filaments (head arrow), MPXV particles (thin arrow), nuclei (N), cell membrane (CM), cytosol (C). Scanning electron microscopy (SEM) images.

The replicative process takes place in the perinuclear space in a granular and fibrous matrix (viral factory) surrounded by mitochondria and RER cisterns, where numerous empty vesicles and particles in formation with different maturation profiles can be seen (Figures 3A–E, 4A–F, 5A–E, Supporting Information Video S2). The interaction of MPXV particles with the cellular cytoskeleton was observed throughout the cytosolic region, underscoring the virus's advanced capacity to commandeer this essential intracellular transport system. This interaction enables MPXV to navigate the cytoplasm efficiently, a process essential for its replication and spread. By sequestering components of the cytoskeleton, including microtubules and actin filaments, MPXV can leverage the motor proteins that traverse these structures, thus facilitating its movement toward replication sites and enhancing its infectivity (Figure 6A–F).

### 3.4 Release of MPXV Particles

**Figure 7:**
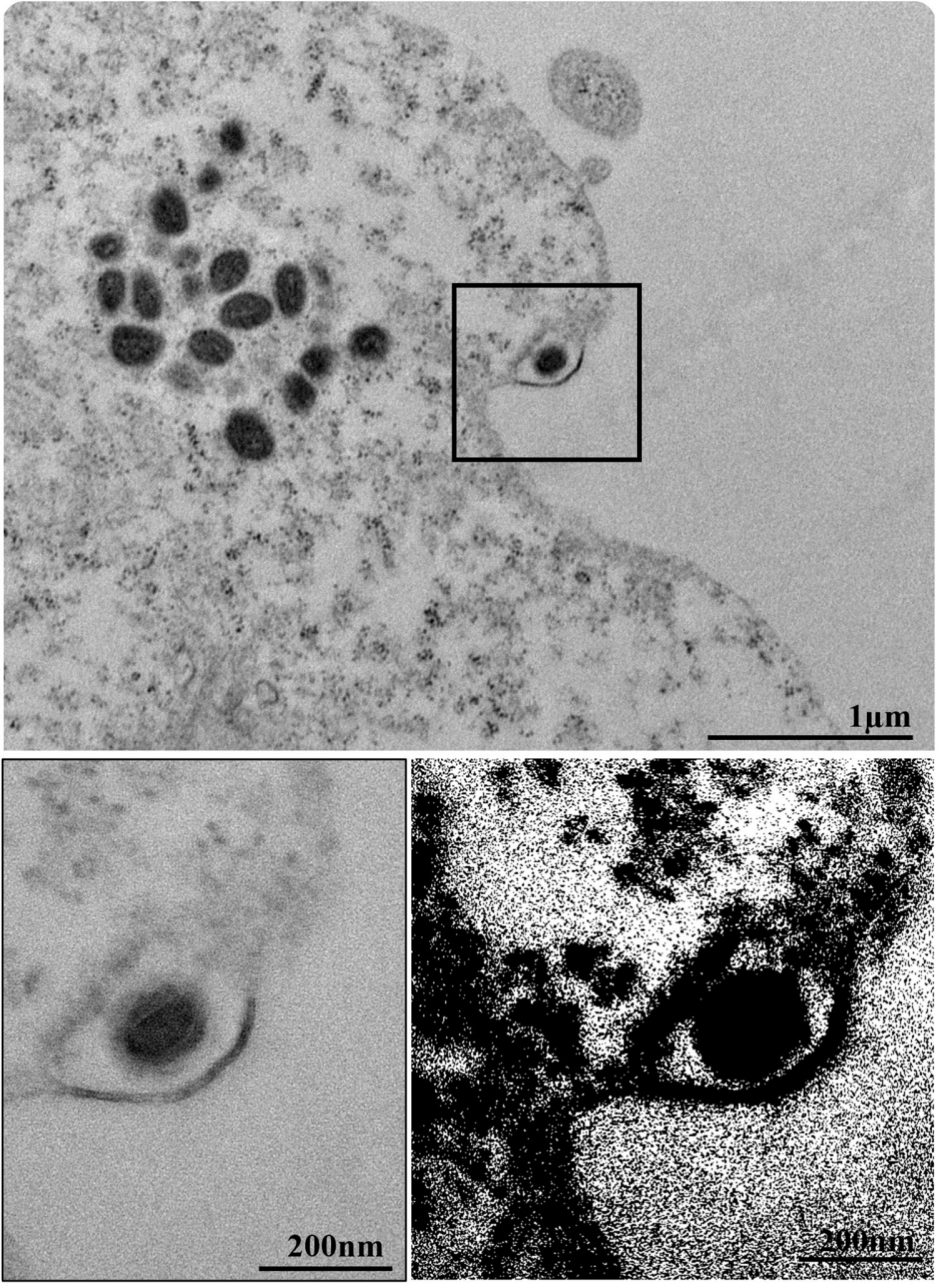
Extrusion of monkeypox virus (MPXV) particle by exocytosis (marked area). MPXV particles (arrow), cytosol (C). Transmission electron microscopy (TEM) images.

**Figure 8:**
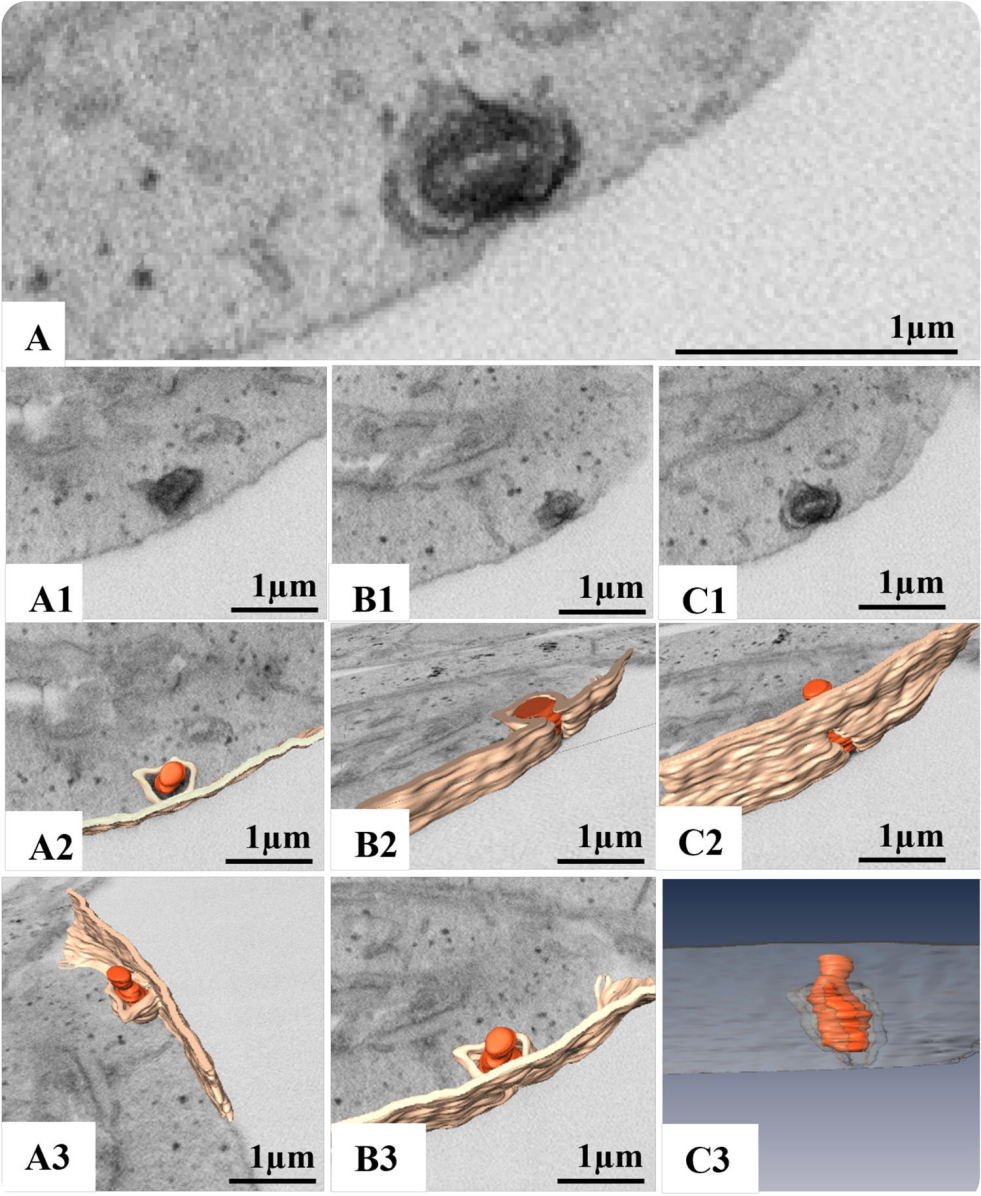
3D Modeling of release of monkeypox virus (MPXV) particles by exocytosis. (A, A1, B1, C1) FIB‐SEM image of the block surface showing the extrusion of the MPXV virus particle. (A2, B2, C2, A3, B3) Combination of a block surface image and the 3D model of structures of interest at different stages from different angles. (C3) 3D model. Orange (MPXV particles), light brown (cell membrane). MPXV particles (arrow). A, A1, B1, C1: FIB‐SEM. See video in Supporting Information (Video S3).

In our analyses employing TEM, FIB‐SEM and three‐dimensional modeling, we observed that the mechanism by which MPXV particles are released is through exocytosis. Our observations consistently revealed the presence of virions within vesicles positioned at the cell periphery, suggesting a highly coordinated mechanism facilitating viral egress (Figures 7 and 8A–C, Supporting Information [Video S3]). Importantly, within a dataset comprising 90 images obtained by MET of infected cells, we identified 13 instances of virion extrusion through exocytosis. This notable finding underscores the efficiency of the exocytic pathway in mediating the release of MPXV particles, highlighting the virus's remarkable capacity to exploit host cellular machinery for effective dissemination.

**Figure 9:**
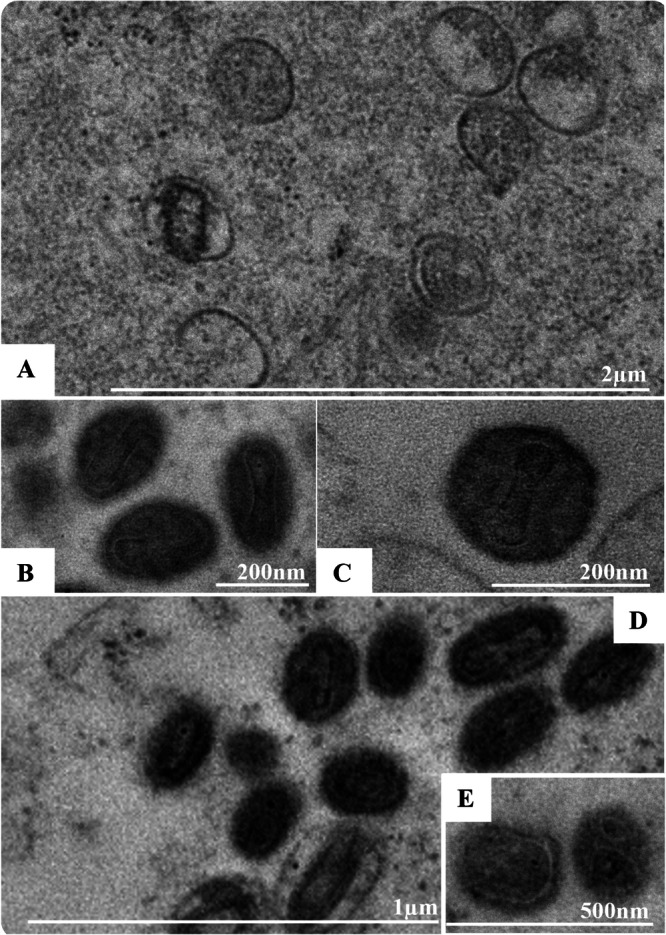
Monkeypox virus (MPXV) particles morphology. (A) Immature particles in different stages: (I) Particles with little electron‐dense granular material in their lumen encircled by a discontinued double membrane (black arrow), (II) Particles with little electron‐dense granular material in their lumen encircled by a double membrane (white arrow), (III) Particles with more electron‐dense and concentrated material in their lumen and encircled by a double membrane (red arrow). (B–E) Mature particles with spherical and brick‐shaped morphology. Transmission electron microscopy (TEM) images.

### 3.5 MPXV Morphology and Diameter

SEM and TEM analyses showed different morphological profiles of immature and mature MPXV particles. The immature particles had little electron‐dense granular material in their lumen and sometimes more electron‐dense and concentrated material, in both cases encircled by a double membrane; at an early stage of maturation, the double membrane was discontinued (Figure 9A). The virions had a pleomorphic morphology ranging from spherical to brick‐shaped (Figure 9B–E). The diameter of the virion ranged from 200 to 500 nm.

## 4 Discussion

There is a consensus that MPXV is a potent inducer of CPE in vitro, leading to massive cell destruction in a few days. Damage has already been observed in different cell lines from both non‐human primates and cells of human origin [33, 34, 35, 36]. The differences then focus on the time taken to observe changes and the range of destruction in the cells. The first signs of change were recorded as early as 36 h after infection in Vero‐E6 cells [37] and from 48 to 72 h in Vero cells [27, 28, 29, 30, 31, 32, 33, 34, 35, 36, 37, 38]. The peak of the effect observed also varied between studies and could occur after three [39], four [37], five [40], or six [27] days post‐infection. Cell rounding, detachment and death, and plaque formation were observed [27, 38, 41, 42, 43]. Schepis et al. [44] demonstrated in vitro that the vaccinia virus changes the cell shape, and that pattern coincides with the rearrangements of the cell's cytoskeleton. At the ultrastructural level, in a previous study carried out by our group, we reported electron‐dense ribosomes, numerous vesicles, mitochondrial swelling and vacuolation, pyknotic nuclei, and altered chromatin profile [27]. The cellular alterations described here corroborate those observed in this study (Figure 1).

In histopathological studies from biopsies of lesions of human cases, inflammatory infiltration in the tissue, ballooning degeneration, and tissue necrosis are frequently reported [45, 46]. The pattern of findings did not change based on the anatomical site of the sample collection [46]. There is no clear correlation between in vitro findings, histopathological, and ultrastructural studies and the severity of the clinical condition.

The 2022 outbreak of MPXV highlighted the importance of dermatological manifestations in its diagnosis, necessitating its inclusion in dermatopathology diagnostics. Studies conducted by Moltrasio et al. [25] examined skin biopsies from patients confirmed positive for MPXV DNA to investigate the histopathological and microscopical characteristics of MPXV‐related cutaneous lesions. Key findings included epidermal necrosis with non‐viable keratinocytes and a “shadow cell” appearance, alongside involvement of hair follicle structures in some cases. Significant cytopathic changes were noted, such as ballooning keratinocytes, Guarnieri bodies, and a ground glass appearance of nuclei, combined with a dense inflammatory infiltrate marked by neutrophil exocytosis. TEM revealed viral aggregates in keratinocyte cytoplasm without nuclear involvement, and viral particles were also detected in mesenchymal cells, albeit in smaller quantities. This research expands the understanding of MPXV histology and microscopy, providing critical insights for future exploration of this emerging pathogen and related Orthopoxvirus infections.

Regarding the replicative cycle, poxviruses present different mechanisms of entry into the cell, such as direct fusion with the cell membrane [47], through reduction of endosomal pH [48], and actin‐dependent macropinocytosis [49]. In our study, we were able to document the entry of MPXV by endocytosis (Figure 2). For particle assembly, it has been known that mature and immature viral particles coexist in the cytoplasm, with the immature being the larger ones [50]. One study also observed a predominance of mature particles [43]. However, in our research, this marked difference was not observed.

Furthermore, as previously demonstrated by Witt and collaborators [43], MPXV infection in Vero cells recruits RER cisternae and reorganizes these structures to form mini‐nuclei. This recruitment also attracted mitochondria to the region, which began to have a more electron‐dense, granular, or fibrillar appearance. The first type is directly linked to acquiring the core for assembly into the viral particle. The second has not yet been proven to be associated with any stage of MPXV morphogenesis [43]. Our analysis showed that part of the synthesis of MPXV occurs in a granular and filamentous matrix surrounded by mitochondria and RER cisterns (Figures 3, 4, 5), as observed by Witt and collaborators [43].

Another point that caught our attention was the interaction of the MPXV particles with the cytoskeleton (Figure 6). In our analysis, the particles were observed to adhere to cytoskeleton filaments from the perinuclear space to the cell periphery. This allows us to infer that these filaments are associated with the transport of these particles toward the extracellular environment. Here, we document the first images obtained by SEM of the interaction of MPXV with cytoskeletal filaments.

Several in vitro studies using light microscopy with the vaccine virus show the importance of the cytoskeleton in the extrusion of viral progeny [51, 52], confirming our findings. Greber and Way demonstrated that the Vaccinia virus uses microtubules to move to the periphery of the cell, given that the large size of the particles would prevent them from moving through diffusion [51]. This displacement through microtubules depends on some mechanisms, including the viral protein F12 [50]. It has also been described that the virus can move through actin polymerization at the tips of actin tails before extending to neighboring uninfected cells [53, 54]. This polymerization is also involved in the process of extrusion of the viral particle from the invaginations in the plasma membrane [55]. This process makes it possible to propel these particles through the external surface, ignoring neighboring infected cells and directing them toward noninfected ones. It would explain the rapidity and amplitude of viral dispersion in monolayer cells observed in vitro [56].

Our analysis by TEM and 3D electron microscopy showed that the release of the MPXV particle occurs by exocytosis, which differs from that observed for other poxviruses, such as the vaccinia virus. In vitro studies with the vaccinia virus report that the release of the viral progeny is done by cell lysis and that virus particles have been observed being transferred to adjacent cells through cytoplasmic prologues resulting from actin polymerization [56, 57].

Furthermore, as MPXV is quite pleomorphic, the significant variability in particle size was already expected. In previous work, particles were reported whose size was in the ranges of 140 to 260 nm [43], 150 to 200 nm [37], 200 to 250 nm [40], 200 to 300 nm [39], 250 to 300 [27], and 256.1 to 361.1 [43]. It is possible to observe that, in all cases, the largest and smallest particles observed always had less than 150 nm difference in size. In our study, however, we observed a variation of 300 nm between the smallest and largest particles, in addition to finding particles measuring up to 500 nm, which is rarely observed in other studies.

Finally, it is important to emphasize the need for expanding ultrastructural studies on MPXV. Despite the longstanding significance of this virus, it has historically been understudied, with limited availability of STEM or TEM images that elucidate details of its replicative cycle. Most existing knowledge is derived from broader research conducted on similar poxviruses, such as Vaccinia virus. However, it is crucial to recognize that viruses within the same family are not identical in all respects. In this context, the studies we conducted using scanning microscopy and 3D reconstruction represent the first presentations in the literature regarding the MPXV. The data from our study, while supporting findings from previous research, also contributes new insights into MPXV morphogenesis. Nonetheless, many specific characteristics of MPXV remain to be elucidated.

## 5 Conclusion

The findings from our study underscore the critical role of TEM in advancing understanding of MPXV. TEM has revealed detailed ultrastructural changes and interactions between MPXV and host cellular components, providing insights that are not possible with other techniques. These discoveries enhance our knowledge of the virus's replication and cytopathogenesis, contributing to the broader field of virology and aiding in the development of targeted therapies and preventative measures against MPXV infections.

## Author Contributions

**Debora Ferreira Barreto‐Vieira:** conceptualization, formal analysis, funding acquisition, investigation, methodology, project administration, resources, writing—original draft, writing—review and editing. **Milene Dias Miranda** and **Ortud Monika Barth:** formal analysis, investigation, methodology, writing—original draft, writing—review and editing. **Marcos Alexandre Nunes da Silva**, **Andressa Santos de Almeida**, **Ana Luisa Teixeira de Almeida**, **Derick Mendes Bandeira**, **Vivian Neuza S. Ferreira**, **Alice Santos Rosa**, **Wendell Girard‐Dias**, and **Bráulio Soares Archanjo:** formal analysis, investigation, methodology and review. All authors have read and agreed to the published version of the article.

## Conflicts of Interest

The authors declare that the research was conducted in the absence of any commercial or financial relationships that could be construed as a potential conflicts of interest. The authors declare no conflicts of interest.

## Supporting information


**Video S1:** 3D Modeling of MPXV particles' entry by endocytosis. Combination of a FIB‐SEM image sequence and the 3D model presenting the entry of the MPXV particle. Orange (MPXV particles), light brown (cell membrane).

**Video S2:** 3D Modeling of the replicative complex of MPXV in Vero cells. Three‐dimensional reconstruction showing different angle views of cellular compartments involved in MPOXV replication. Dark blue (nucleus), orange (virion), green (mitochondria), yellow (rough endoplasmic reticulum), violet (empty vesicles), purple (immature MPXV particles), light blue (viral DNA).

**Video S3:** 3D Modeling of release of MPXV particles by exocytosis. Combination of a FIB‐SEM image of the block surface and 3D model showing the extrusion of the MPXV virus particle at different stages from different angles. Orange (MPXV particles), light brown (membrane cell).

## Data Availability

The data that support the findings of this study are available on request from the corresponding author. The data are not publicly available due to privacy or ethical restrictions.
